# Laparoscopic vs. open inguinal hernia repair outcomes in patients aged 65 years and older

**DOI:** 10.3389/fsurg.2025.1610358

**Published:** 2025-12-08

**Authors:** Abdulkarem Al-Sharabi, Yang Xu, Abdullah Al-Danakh, ZhaoHui Xu, Abdullah Al-Sharabi, HaoNan Kang, GuoYi Wen, Yan Shan, BoXin Qu, Shuai Shao, YanYing Ren, Juyeong Lee, Fan Zhang, Xin Chen

**Affiliations:** 1 Department of Hernia and Colorectal Surgery, The Second Hospital of Dalian Medical University, Dalian, China; 2 Department of Urology, First Affiliated Hospital of Dalian Medical University, Dalian, China; 3 School of Optometry, Jiangxi Medical College, Nanchang University, Nanchang, Jiangxi, China

**Keywords:** elderly patients, inguinal hernia surgery, laparoscopic repair, open repair, postoperative outcomes

## Abstract

**Background:**

Inguinal hernia affects approximately 27% of men and 3% of women, with prevalence rising markedly after age 65 due to weakened abdominal walls and comorbidities such as chronic obstructive pulmonary disease and benign prostatic hyperplasia, which increase intra-abdominal pressure. Standard treatment involves surgical repair using either open or laparoscopic techniques. However, the optimal approach for elderly patients remains debated due to higher comorbidity rates and unique physiological considerations. This study compares outcomes of laparoscopic and open inguinal hernia repair in patients aged ≥65 years to support evidence-based surgical decision-making.

**Methods:**

We conducted a retrospective analysis of 384 patients aged ≥65 years who underwent inguinal hernia repair at the Second Affiliated Hospital of Dalian Medical University between 2020 and 2022. Patients were classified into laparoscopic (*n* = 197) and open (*n* = 187) repair groups. Demographics, operative details, and postoperative outcomes were compared using chi-square tests, Mann–Whitney *U* tests, and multivariable logistic regression (SPSS v27), with statistical significance set at *p* < 0.05.

**Results:**

The laparoscopic cohort was younger [median 70 (65–91) vs. 75 (65–98) years; *p* < 0.001], had a higher prevalence of bilateral hernias (25.9% vs. 4.3%; *p* < 0.001), and smaller hernia sacs [2.50 (1–5) cm vs. 3 (1–5) cm; *p* < 0.001]. Although operative time was longer for laparoscopy [76 (40–120) vs. 60 (40–115) minutes; *p* < 0.001], it was associated with fewer postoperative complications, shorter hospital stays, and lower chronic pain rates (all *p* < 0.01). Multivariable logistic regression identified open repair as an independent risk factor for complications (OR = 0.359; *p* = 0.012) and chronic pain (OR = 0.258; *p* = 0.009). Patients in the laparoscopic group also resumed normal activities sooner.

**Conclusion:**

Although laparoscopic inguinal hernia repair requires a longer operative time, it offers superior outcomes in elderly patients, including fewer complications, reduced chronic pain, and faster recovery. Careful patient selection based on age, hernia characteristics, and overall health is essential; however, when feasible, laparoscopy should be considered the preferred approach to optimize outcomes in this population.

## 1 Introduction

An inguinal hernia is an abdominal wall defect occurring in the groin region (1, 2) and is one of the most common surgical conditions worldwide, with approximately 20 million repairs performed annually (3). The prevalence increases with age, from 0.25% at 18 years to 4.2% between 75 and 80 years (4). In the United States, more than 800,000 inguinal hernias are repaired each year (5, 6). In China, 14.19% of the population is aged ≥65 years, slightly above the global average (7) and hernia incidence is increasing. Between 1990 and 2019, India, China, and Brazil accounted for 39% of global cases, with China reporting 1.95 million cases during this period (8). Given this increasing prevalence in elderly populations, understanding the optimal surgical approach becomes crucial, as patient age and associated comorbidities significantly influence treatment decisions and outcomes.

Factors associated with an increased risk of developing an inguinal hernia include male sex, advanced age, low body mass index, prior prostate surgery, systemic connective tissue disorders, previous radiation therapy, and a family history of hernias, as indicated by epidemiological studies (9). The first incidence peak occurs during infancy, while the second is seen in the fourth decade of life (10). Men have a higher lifetime prevalence of inguinal hernias than women, ranging from 27%–43% (11). It is also more common in elderly adults due to diminished abdominal wall strength and other medical conditions that increase intra-abdominal pressure, notably chronic obstructive pulmonary disease and benign prostatic hyperplasia (12).

Patients with inguinal hernia typically present with a groin bulge that may gradually enlarge over time (9). While urgent surgical intervention is required in certain situations, most cases are managed electively (13). Emergency surgery is indicated when there is sudden, severe abdominal pain suggestive of incarceration (14). Compared with younger individuals, elderly patients experience higher rates of postoperative mortality and morbidity (15, 16). Although older adults with comorbidities may have minimal symptoms and relatively low short-term risk, the optimal timing for intervention remains uncertain (17). Some researchers recommend early elective repair in elderly patients with comorbidities, whereas others support close monitoring in young, otherwise healthy individuals (18, 19). Notably, a Swedish study reported that the mortality rate for elective hernia repair is low, even among patients over 80 years of age (0.58%) (20). Surgical repair remains the most effective treatment for inguinal hernia (21).

The choice between open repair and laparoscopic mesh repair depends on patient characteristics, anticipated operative time, and complication risk (4). Over the past two decades, numerous studies have compared these approaches (8). The Lichtenstein open mesh repair is a well-established technique with a recurrence rate below 1% (22), but chronic pain remains a notable complication, affecting 5%–10% of patients and reported in up to 63% of cases (23). Since the introduction of laparoscopic techniques by Watson et al. in 1993 (24). Debate has continued regarding their advantages and disadvantages, particularly in elderly patients. Laparoscopic inguinal hernia repair (LIHR) has gained popularity worldwide due to advances in minimally invasive surgery, offering reduced wound complications, lower recurrence rates, and faster recovery (25–27). However, open repair remains necessary in patients with coagulopathy, significant preperitoneal adhesions, or extraperitoneal hemorrhage (28). In elderly patients with poor cardiopulmonary reserve or intolerance to general anesthesia, open repair under regional anesthesia is generally preferred (29).

This study seeks to determine the optimal surgical approach for managing inguinal hernias in patients aged ≥65 years. In surgical research, outcomes refer to measurable clinical results following an intervention, including postoperative complications, chronic pain, length of hospital stay, recovery time, and recurrence rates (8, 11, 30). Evaluating these outcomes provides objective evidence to guide the selection between open and laparoscopic inguinal hernia repair, particularly in elderly patients. Therefore, we conducted a retrospective analysis to compare the clinical outcomes of laparoscopic and open inguinal hernia repair in this population.

## 2 Material and methodology

### 2.1 Study design and participants

This study was performed as a retrospective comparative analysis. A total of 384 patients with inguinal hernia, including both indirect and direct types, underwent elective surgical intervention. Patients admitted to the Department of Colorectal and Hernia Surgery at the Second Affiliated Hospital of Dalian Medical University between 2020 and 2022 were selected. The research protocol received approval from the Medical Ethics Committee of the Second Affiliated Hospital of Dalian Medical University and complied with the ethical principles established in the Declaration of Helsinki. The retrospective design of the study removed the necessity for written informed consent from the patients involved.

### 2.2 Study population and inclusion & exclusion criteria

All patients were categorized who underwent elective inguinal hernia repair at the Second Affiliated Hospital of Dalian Medical University between January 2020 and June 2022 into two groups: group A (open approach) and group B (laparoscopic approach).

**Figure 1:**
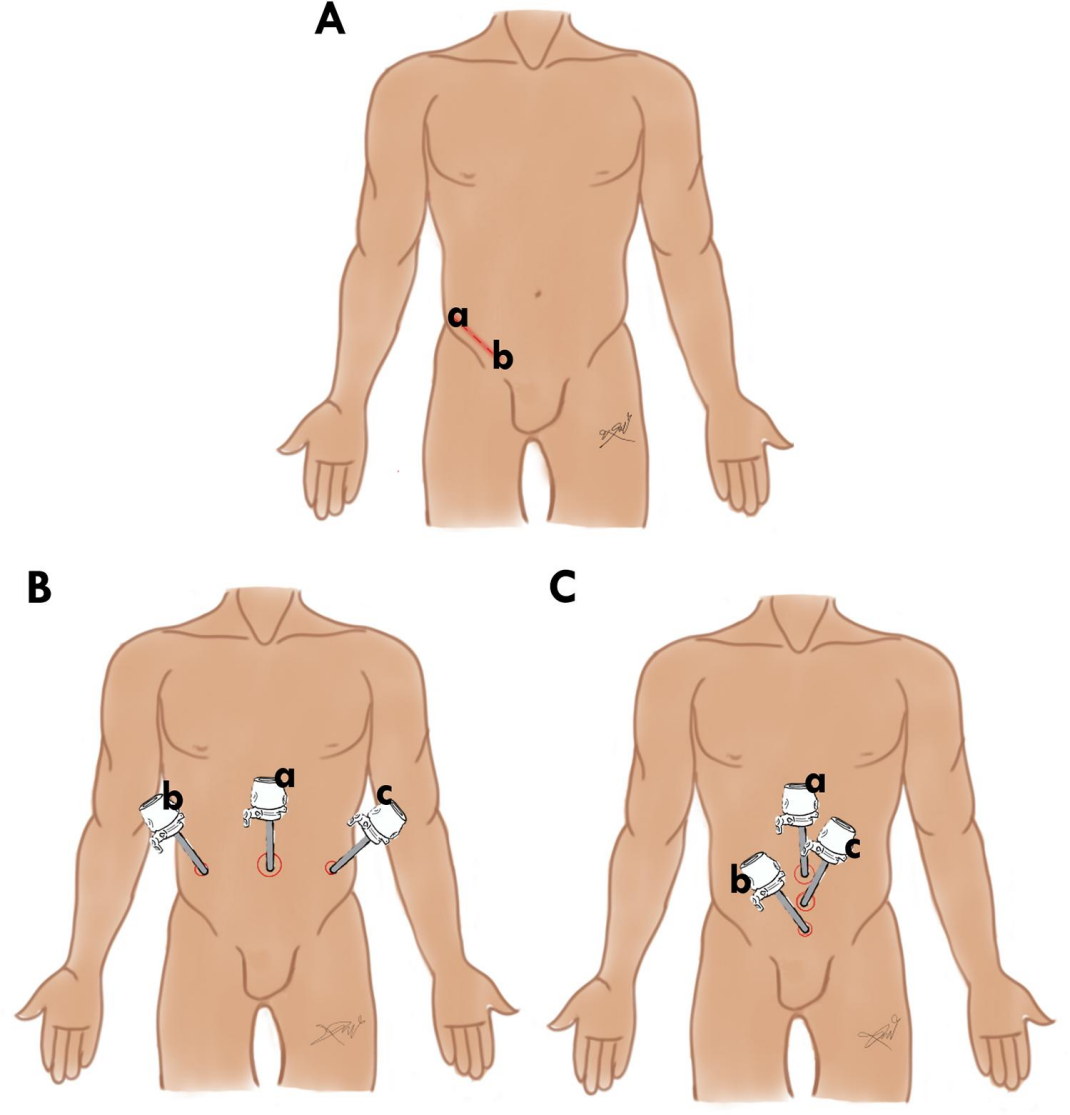
Surgical approach of inguinal hernia repair: **(A)** incision line of open inguinal hernia approach from **(a)** the anterior superior iliac spine to the **(b)** pubic tubercle 5 cm. **(B)** Laparoscopic transabdominal preperitoneal approach, **(a)** 10 mm incision for trocar of the camera, **(b,c)** Two 5 mm incisions for working trocars. **(C)** Laparoscopic totally extraperitoneal approach, **(a)** 10 mm incision for trocar of the camera, **(b,c)** Two 5 mm incisions for working trocars.

The following inclusion criteria were adopted:

- Patients who had to be 65 years old or older
- Patients who had to have undergone either open inguinal hernia repair or laparoscopic with both types (TAPP, TEP) (Figure 1).
- Patients who had a direct or indirect inguinal hernia.

The exclusion criteria included the following:

- Individuals under the age of 65 years.
- Cases of recurrent inguinal hernia.
- Emergencies involving incarcerated or strangulated hernias.
- Cases where the surgical procedure shifted from laparoscopic to open inguinal hernia repair during surgery.
- An additional type of inguinal hernia, such as a femoral hernia, etc.

**Figure 2:**
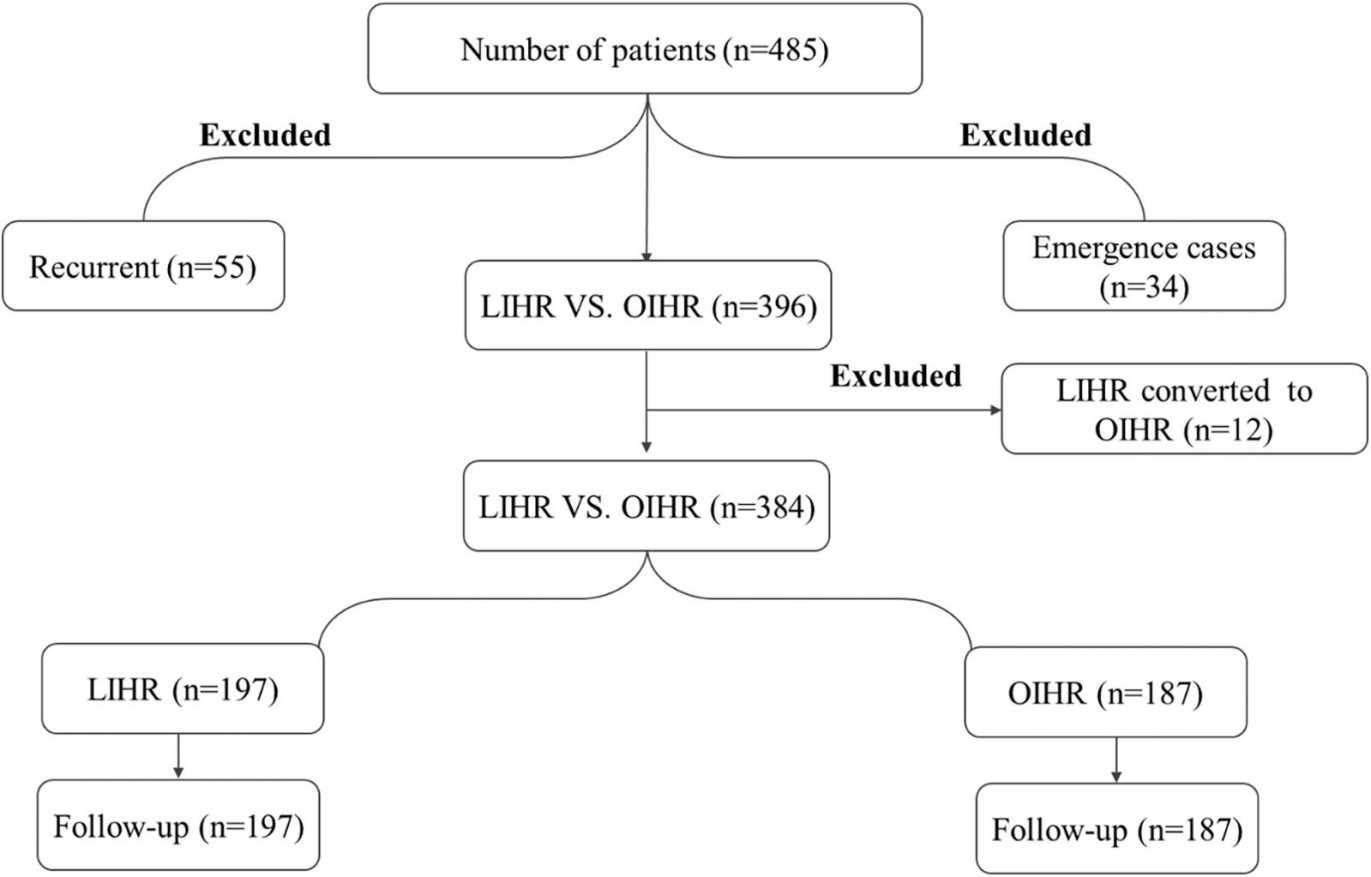
Study flowchart for inguinal hernia patients in both open and laparoscopic surgical groups: n, simple size number; LIHR, laparoscopic inguinal hernia repair; OIHR, open inguinal hernia repair.

In the beginning, we retrieved a total of 485 patients who participated in this study; however, after applying the inclusion and exclusion criteria, only 384 individuals who met our criteria were included in the analysis. Group A (Open approach) consisted of 187 patients, whereas Group B (Laparoscopic approach) comprised 197 patients (Figure 2).

### 2.3 Data collection

We abstracted data from the electronic medical records using a standardized form. Baseline variables included age, sex, body mass index, tobacco and alcohol use, and American Society of Anesthesiologists (ASA) classification, which was dichotomized as grades I–II vs. III–IV. Comorbidities were recorded individually and comprised coronary artery disease, diabetes mellitus, hypertension, congestive heart failure, atrial fibrillation, chronic obstructive pulmonary disease, cerebrovascular disease, and benign prostatic hyperplasia. Hernia-related characteristics included site (unilateral/bilateral), type (indirect/direct), overall hernia size, and sac size.

Perioperative variables encompassed surgical approach [open vs. laparoscopic (TAPP or TEP)], type of anesthesia, operative time, mesh use, estimated blood loss, intraoperative intravenous fluid volume, and urine output. Postoperative endpoints included the occurrence of complications, specifically surgical-site infection, seroma, hematoma, and delirium, each recorded as present or absent. We also captured length of hospital stay, the presence of chronic pain, time to return to normal activities or work, and hernia recurrence.

All patients were followed at approximately 1, 6, and 12 months after surgery, either in outpatient clinic visits or by telephone interview. At each contact, we ascertained recurrence status, chronic pain, and return-to-activity timing. For missing data, we applied a complete-case approach: patients lacking information for a given primary endpoint (e.g., no 12-month recurrence assessment) were excluded from the analysis of that endpoint but retained in analyses for which their data were complete. No statistical imputation was performed.

## 3 Statistical analysis

Data analysis was conducted using IBM SPSS version 27 (IBM Corp., Armonk, NY) and GraphPad version 8. Continuous variables were analyzed by calculating medians, while categorical variables were assessed using frequencies and percentages. The distribution of numerical variables was assessed using the Shapiro–Wilk test, indicating an abnormal distribution and confirming the appropriateness of non-parametric testing. Medians and non-parametric independent tests, specifically the Mann–Whitney *U* test, were utilized for analysis. We utilized chi-square analysis or Fisher's exact test to compare categorical data, contingent upon the number of participants in each category. We conducted univariate and multivariate binary logistic regression analyses to identify predictive factors. A *p*-value less than 0.05 was deemed statistically significant in the statistical analyses.

## 4 Results

### 4.1 Clinical characteristics of patients

**Figure 3:**
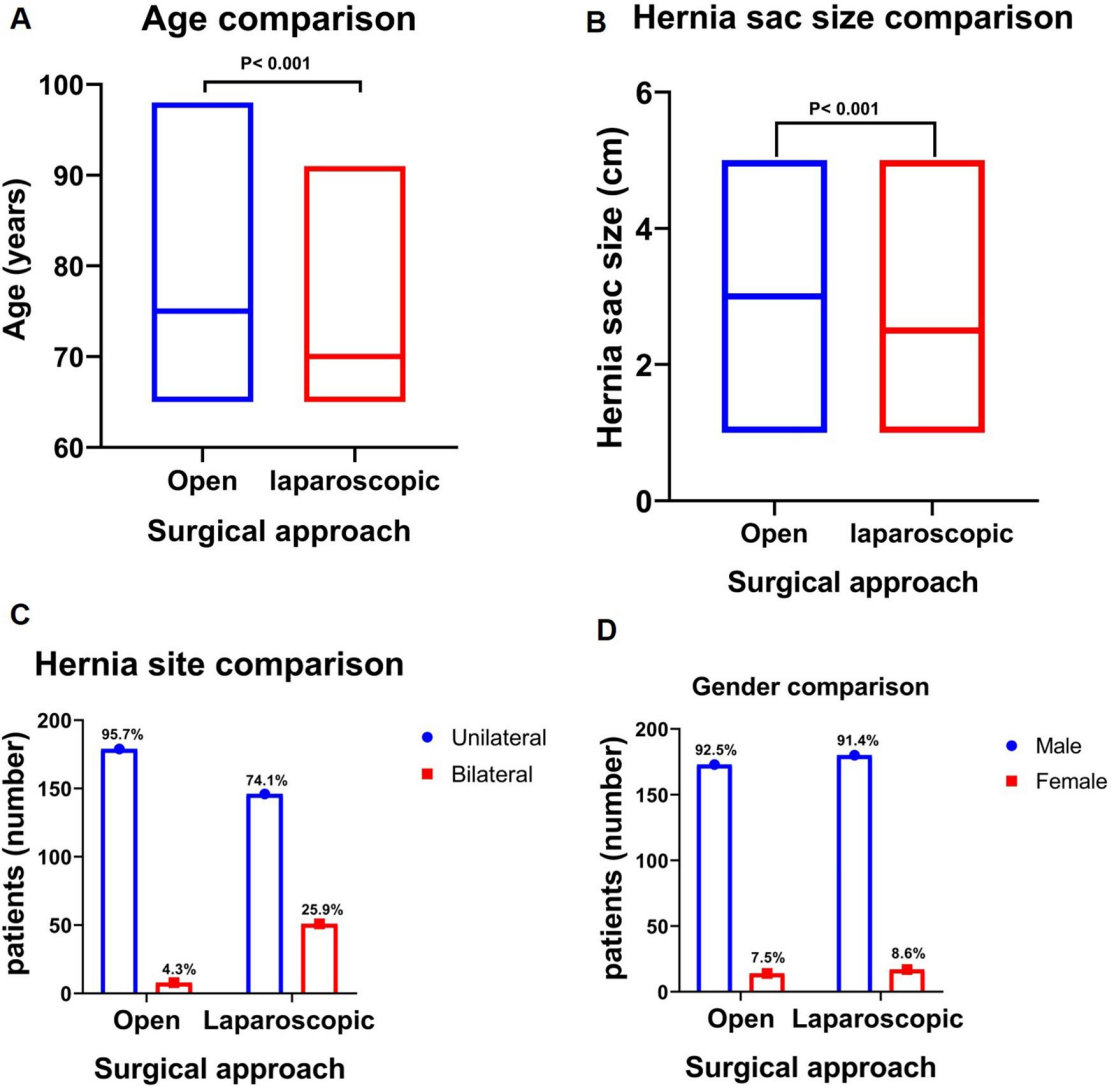
Clinical characteristics of laparoscopic vs. open repair for inguinal hernia patients: **(A)** Age in years: The median age of the laparoscopic repair group was substantially lower than that of the open repair group (*P* < 0.001). **(B)** Number of gender types in each approach: The gender distribution was comparable between the open and laparoscopic repair groups. **(C)** Hernia site (Unilateral/Bilateral): The laparoscopic group exhibited a higher percentage of bilateral hernias (*P* < 0.001). **(D)** Hernia sac size (cm): The median hernia sac size was larger in the open repair group than in the laparoscopic group (*P* < 0.001).

This study enrolled 384 patients who underwent elective inguinal hernia surgery. Of these, 187 (48.6%) underwent open surgery, while 197 (51.4%) received laparoscopic procedures. The median age was significantly lower in the laparoscopic hernia repair group compared to the open hernia repair group (*P* < 0.001) (Figure 3A). No statistically significant distinction was observed in gender treated with either open or laparoscopic hernia repair groups, in which the male gender represented the majority of cases in both groups (92.5% and 91.4%, respectively, *P* = 0.681) (Figure 3B). The distribution of hernia sites showed a significant difference between unilateral and bilateral hernias, with a higher prevalence of bilateral hernias in the laparoscopic hernia repair group (25.9% vs. 4.3%, *P* < 0.001) (Figure 3C). By studying hernia type between the two surgical approaches, there was no significant difference between indirect and direct hernias. Interestingly, when we compared the hernial sac size there was a statistically significant difference between the two surgical methods, the median size was 3 cm in the open vs. 2. 5 cm in the laparoscopic group (*P* = 0.001) (Figure 3D). The distribution of comorbidities was similar between groups, with no significant differences observed for cardiovascular, metabolic, or pulmonary conditions (Table 1). ASA classification showed comparable distributions (74.3% vs. 73.6% for grades I/II, *P* = 0.871). Detailed clinical characteristics are in (Table 1).

**Table 1:** Clinical characteristics of inguinal hernia patients who underwent open or laparoscopic repair.

Variables	Open (Total = 187)	Laparoscopic (Total = 197)	*P*-value
Gender			
Male	173 (92.5%)	180 (91.4%)	0.681
Female	14 (7.5%)	17 (8.6%)	
Age (years), Median (IQR)	75 (65–98)	70 (65–91)	**<0** **.** **001**
Hernia site			
Unilateral	179 (95.7%)	146 (74.1%)	**<0** **.** **001**
Bilateral	8 (4.3%)	51 (25.9%)	
Hernia type			
Indirect	154 (82.4%)	159 (80.7%)	0.679
Direct	33 (17.6%)	38 (19.3%)	
Hernia size (cm), median (IQR)	2.50 (1–5)	2.50 (1–5)	0.522
Hernia sac size (cm), median (IQR)	3 (1–5)	2.50 (1–5)	**<0** **.** **001**
ASA			
(I/II)	139 (74.3%)	145 (73.6%)	0.871
(III/IV)	48 (25.7%)	52 (26.4%)	
Coronary artery disease			
No	170 (90.9%)	179 (90.9%)	0.987
Yes	17 (9.1%)	18 (9.1%)	
Diabetes			
No	160 (85.6%)	167 (84.8%)	0.828
Yes	27 (14.4%)	30 (15.2%)	
Hypertension			
No	158 (84.5%)	173 (87.8%)	0.345
Yes	29 (15.5%)	24 (12.2%)	
Congestive heart failure			
No	183 (97.9%)	192 (97.5%)	1.000
Yes	4 (2.1%)	5 (2.5%)	
Atrial fibrillation			
No	179 (95.7%)	193 (98.0%)	0.352
Yes	7 (3.7%)	4 (2.0%)	
Cerebrovascular disease			
No	174 (93.0%)	187 (94.9%)	0.439
Yes	13 (7.0%)	10 (5.1%)	
COPD			
No	181 (96.8%)	190 (96.4%)	0.852
Yes	6 (3.2%)	7 (3.6%)	
Benign prostatic hyperplasia			
No	21 (11.2%)	34 (17.3%)	0.092
Yes	166 (88.8%)	163 (82.7%)	
BMI (kg/m^2^), Median, IQR	24.2 (18.6–32)	24.2 (18.6–32)	0.950
Smoking			
No	159 (85.0%)	164 (83.2%）	0.634
Yes	28 (15.0%)	33 (16.8%)	
Alcohol			
No	148 (79.1%)	154 (78.2%)	0.816
Yes	39 (20.9%)	43 (21.8%)	
Mesh use			
Yes	187 (100%)	197 (100%)	-

ASA, American society of anesthesiologists; COPD, chronic obstructive pulmonary disease; BMI, body mass index; IQR, interquartile ranges.

Bold values indicate *P* < 0.05.

### 4.2 Operation and postoperative characteristics

**Figure 4:**
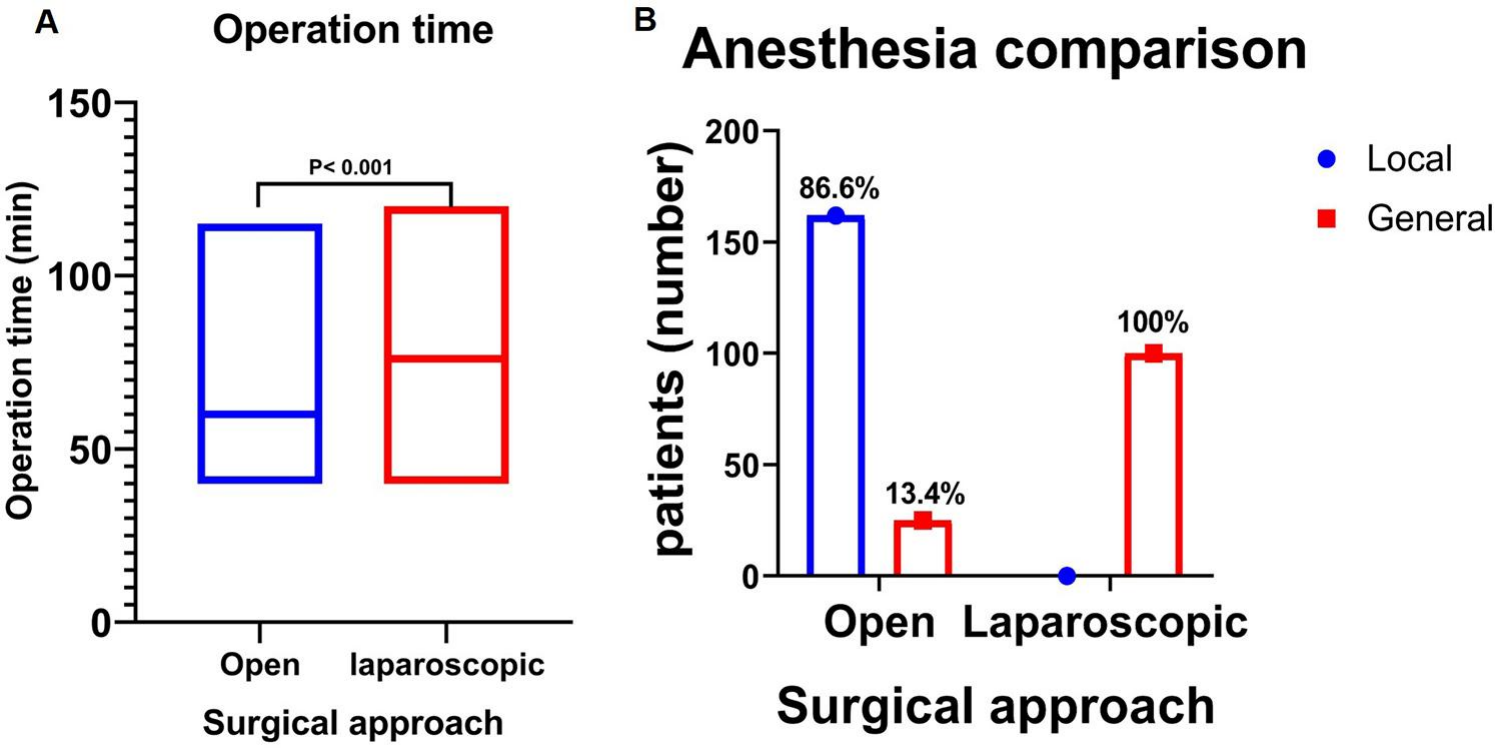
Comparison of operation time and anesthesia types according to surgical approach (open vs. Laparoscopic): **(A)** Operation Time (min): There was a prolonged operation time in the laparoscopic hernia repair group than in the open group. **(B)** Comparison of Anesthesia: The open hernia repair group primarily employed local anesthesia, whereas the laparoscopic group exclusively employed general anesthesia. min, minutes.

In the evaluation of operation-related variables, we found that the laparoscopic hernia repair group had a significantly longer operation time of 76 min vs. 60 min in the open group (*P* < 0.001) (Figure 4A). For those undergoing laparoscopic surgery, they receive (100%) general anesthesia, while in open surgery cases only (13.4%) have general anesthesia (*P* < 0.001) (Figure 4B). Detailed operative items are summarized in the (Table 2).

**Table 2:** Comparisons of operation data between open vs. laparoscopic repair for inguinal hernia patients.

Variables	Open	Laparoscopic	*P*-value
Type of anesthesia			**<0** **.** **001**
Local (regional or spinal)	162 (86.6%)	0 (0.0%)	
General	25 (13.4%)	197 (100%)	
Operation time (minutes), median (IQR)	60 (40–115)	76 (40–120)	**<0** **.** **001**
Blood loss (ml), median (IQR)	5 (2–50)	5 (2–30)	0.514
Intraoperative intravenous fluid infusion(ml), median (IQR)	900 (600–1,200)	800 (600–1,200)	0.324
Intraoperative urine output(ml), IQR	700 (400–1,100)	700 (400–1,100)	0.940

IQR, interquartile ranges.

Bold values indicate *P* < 0.05.

**Figure 5:**
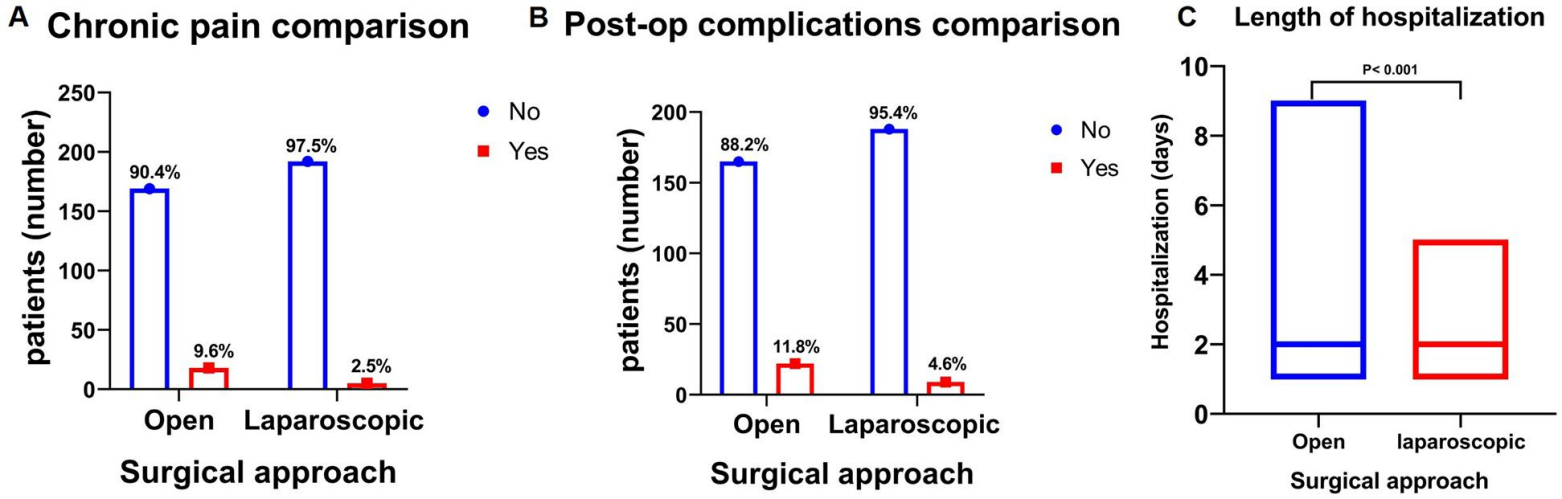
Comparison of postoperative outcomes for open vs. Laparoscopic Approaches. **(A)** Number of patients with chronic pain: The laparoscopic group exhibited a significantly lower incidence of chronic pain than the open repair group (*P* = 0.003). **(B)** Number of patients with postoperative complications: The laparoscopic group exhibited lower postoperative complications (*P* = 0.010). **(C)** Length of hospitalizations (days): The duration of hospitalization was significantly shorter in the laparoscopic group than in the open surgery group (*P* < 0.001).

When we analyze postoperative variables between the two surgical approaches, In comparison to the open group, the laparoscopic group exhibited a substantially lower incidence of chronic pain. (*P* = 0.003) (Figure 5A). Similarly, there were fewer postoperative complications in the laparoscopic group compared to the open group, with statistical significance (*P* = 0.010) (Figure 5B). Furthermore, the median length of hospitalization for the laparoscopic group was shorter compared to the open hernia repair group, which was statistically significant (*P* < 0.001) (Figure 5C). The recurrence rate at one year for two groups was not significant (*P* = 0.718) (Table 3).

**Table 3:** Comparisons of operation outcomes between open vs. laparoscopic repair for inguinal hernia patients.

Variables	Open	Laparoscopic	*P*-value
Chronic pain			
No	169 (90.4%)	192 (97.5%)	**0** **.** **003**
Yes	18 (9.6%)	5 (2.5%)	
Post-op complications			
No	165 (88.2%)	188 (95.4%)	**0** **.** **010**
Yes	22 (11.8%)	9 (4.6%)	
Length of hospitalization (days), median (IQR)	2 (1–9)	2 (1–5)	**<0** **.** **001**
Return to work (days), median (IQR)	30 (23–45)	30 (21–45)	0.144
Recurrence at one year			
No	183 (97.9%)	194 (98.5%)	0.718
Yes	4 (2.1%)	3 (1.5%)	

IQR, interquartile ranges.

Bold values indicate *P* < 0.05.

### 4.3 Potential factors influencing the selection of surgical approach for hernia repair

To further investigate the significance of our comparative analysis findings, we conducted binary univariate logistic regression to identify factors associated with surgical approach selection. The regression analysis revealed several significant predictors that influenced the choice between laparoscopic and open-surgical techniques (Table 4). Subsequent multivariate logistic regression analysis demonstrated that age remained a significant predictor (OR = 0.881, 95% CI: 0.838–0.926, *P* < 0.001), indicating that laparoscopic procedures were more prevalent among younger patients. The hernia site emerged as a strong predictor (OR = 11.588, 95% CI: 3.956–33.944, *P* < 0.001). The size of the hernia sac also significantly influenced surgical choice (OR = 0.620, 95% CI: 0.442–0.872, *P* = 0.006), with smaller hernias being more frequently addressed laparoscopically.

**Table 4:** Univariable and multivariable analysis for hernial repair surgical approaches in inguinal hernia patients.

Variables	Univariable logistic regression			Multivariable logistic regression		
	OR	95% CI	*P*-value	OR	95% CI	*P*-value
Age (years)	0.893	0.862–0.925	<0.001	0.881	0.838–0.926	**<0** **.** **001**
Gender (M/F)	1.167	0.558–2.440	0.681			
Hernia site (unilateral/bilateral)	7.816	3.595–16.994	<0.001	11.588	3.956–33.944	**<0** **.** **001**
Type of hernia (direct/indirect)	1.115	0.666–1.869	0.679			
Hernia sac size (cm)	0.658	0.526–0.822	<0.001	0.620	0.442–0.872	**0** **.** **006**
Hernia size (cm)	1.101	0.866–1.400	0.432			
ASA (I/II) (III/IV)	1.039	0.658–1.639	0.871			
Operation time (minutes)	1.101	1.078–1.124	<0.001	1.116	1.087–1.147	**<0** **.** **001**
Hospitalization (days)	0.644	0.540–0.767	<0.001	0.576	0.437–0.759	**<0** **.** **001**
Post-op complication	0.359	0.161–0.802	0.012	0.173	0.051–0.582	**0** **.** **005**
Chronic pain	0.245	0.089–0.673	0.006	0.155	0.034–0.715	**0** **.** **017**
Recurrence at one year	0.707	0.156–3.204	0.653			
Return to work (days)	0.962	0.920–1.006	0.093			

ASA, American society of anesthesiologists; OR, odd ratio; CI, confidence interval.

Bold values indicate *P* < 0.05.

Operational factors showed significant associations as well. Operation time demonstrated a positive correlation with the laparoscopic approach (OR = 1.116, 95% CI: 1.087–1.147, *P* < 0.001), while the length of hospitalization showed an inverse relationship (OR = 0.576, 95% CI: 0.437–0.759, *P* < 0.001). Post-operative outcomes were also significant predictors, with both post-operative complications (OR = 0.173, 95% CI: 0.051–0.582, *P* = 0.005) and chronic pain (OR = 0.155, 95% CI: 0.034–0.715, *P* = 0.017) showing lower odds in the laparoscopic group (Table 4).

### 4.4 Potential factors for postoperative chronic pain

Further analysis of chronic pain outcomes revealed significant differences between surgical approaches. Patients who underwent laparoscopic hernia repair demonstrated a markedly lower incidence of chronic pain compared to those who received open repair [5 (2.5%) vs. 18 (9.6%), *P* = 0.003] (Table 3). Initial univariable analysis identified a strong association between surgical approach and the development of chronic pain (Table 5).

**Table 5:** Univariable and multivariable analysis for chronic pain in inguinal hernia patients.

Variables	Univariable logistic regression			Multivariable logistic regression		
	OR	95% CI	*P*-value	OR	95% CI	*P*-value
Surgical approach (OIHR vs. LIHR)	0.245	0.089–0.673	0.006	0.258	0.093–0.714	**0** **.** **009**
Age (years)	1.002	0.942–1.067	0.945			
Gender (M/F)	0.502	0.065–3.851	0.507			
Hernia site (unilateral/bilateral)	0.508	0.116–2.226	0.369			
Hernia type (direct/indirect)	0.924	0.304–2.804	0.889			
Hernia sac size (cm)	1.196	0.776–1.842	0.418			
Hernia size (cm)	0.781	0.457–1.336	0.367			
ASA (I/II) (III/IV)	0.581	0.193–1.751	0.335			
Operation time (minutes)	0.979	0.953–1.006	0.126			
Return to work (days)	1.088	1.008–1.175	0.030	1.078	0.997–1.165	**0** **.** **059**

ASA, American society of anesthesiologists; OIHR, open inguinal hernia repair; LIHR, laparoscopic inguinal hernia repair; OR, odd ratio; CI, confidence interval.

Bold values indicate *P* < 0.05.

To establish independent predictors of chronic pain, we conducted a comprehensive multivariable regression analysis. This analysis confirmed that open hernia repair represented an independent risk factor for the development of chronic pain (OR = 0.258, 95% CI: 0.093–0.714, *P* = 0.009). Subsequently, we observed that patients experiencing chronic pain demonstrated significantly delayed return to work times, suggesting that post-operative pain may be a determining factor in recovery and resumption of normal activities (OR = 1.078, 95% CI: 0.997–1.165, *P* = 0.059) (Table 5).

### 4.5 Potential factors for operation time

Analysis of factors influencing operation time revealed several significant associations through univariable analysis. Initial findings indicated that surgical approach, age, hernia site, and type of anesthesia demonstrated significant relationships with operative duration. Subsequent multivariable regression analysis identified two independent predictors of prolonged operation time: hernia size (OR = 1.550, 95% CI: 1.111–2.161, *P* = 0.010) and the presence of hypertension (OR = 0.270, 95% CI: 0.102–0.713, *P* = 0.008) (Table 6).

**Table 6:** Univariable and multivariable analysis for operation time in inguinal hernia patients.

Variables	Univariable logistic regression			Multivariable logistic regression		
	OR	95% CI	*P*-value	OR	95% CI	*P*-value
Surgical approach (OIHR vs. LIHR)	33.357	13.357–86.045	**<0** **.** **001**			
Age (years)	0.929	0.892–0.967	**<0** **.** **001**			
Gender (M/F)	1.392	0.631–3.067	0.412			
Hernia site (unilateral/bilateral)	2.247	1.259–4.010	**0** **.** **006**			
Hernia type (direct/indirect)	1.142	0.642–2.032	0.651			
Hernia sac size (cm)	0.870	0.680–1.113	0.268			
Hernia size (cm)	1.445	1.105–1.889	**0** **.** **007**	1.550	1.111–2.161	**0** **.** **010**
Hypertension	0.322	0.133–0.778	**0** **.** **012**	0.270	0.102–0.713	**0** **.** **008**
ASA (I/II) (III/IV)	1.225	0.737–2.038	0.434			
Anesthesia type (local, regional or spinal/general)	23.488	9.275–59.484	**<0** **.** **001**			

ASA, American society of anesthesiologists; OR, odd ratio; CI, confidence interval.

Bold values indicate *P* < 0.05.

## 5 Discussion

The surgical management of inguinal hernia remains a significant healthcare challenge, with over 20 million procedures performed annually worldwide, representing 75% of all abdominal wall hernias (16). The condition disproportionately affects males (27%) compared to females (3%) (31), with increasing prevalence anticipated as global demographics shift toward an aging population (32). Despite the high frequency of inguinal hernia repair among elderly patients (aged 65 and older) (33), there remains a notable research gap regarding the optimal surgical approach for this demographic. The objective of this investigation is to assess the comparative effectiveness of laparoscopic and open hernia repair in elderly patients.

Our comparative analysis of open and laparoscopic inguinal hernia repair groups revealed several significant findings. Notably, neither group experienced mortality, and laparoscopic intervention demonstrated fewer complications, reduced chronic pain, and faster recovery times, consistent with previous research (8, 29, 34, 35). These advantages likely stem from smaller surgical incisions and improved postoperative wound healing dynamics. Our findings align with earlier studies (36), examining open preperitoneal inguinal hernia repair outcomes.

The study identified significant correlations between open inguinal hernia repair and various parameters, including age, hernia site, hernia sac size, hospital stay duration, and operation time. While laparoscopic repair offers numerous advantages (37), it typically requires longer operative times, reflecting the established learning curve for surgeons adopting these techniques (30). Extended operative duration in laparoscopy may result in elevated expenses attributable to factors such as increased consumption of surgical supplies, prolonged anesthesia duration, and extended hospital admissions. Although laparoscopy typically incurs lower overall costs relative to open surgery due to diminished hospital stays and expedited recovery, an elongated laparoscopic procedure can undermine some of these advantages (38). Although all procedures were performed by the same team of senior surgeons, the extended operation time in the laparoscopic group may be attributed to additional patient preparation and port placement requirements.

Contrary to previous studies highlighting concerns about anticoagulant and thrombolytic medications affecting bleeding tendencies in elderly patients (28, 39, 40). Our analysis showed no significant differences in bleeding or hematoma formation between approaches, possibly due to effective preoperative medication management. Laparoscopic inguinal hernia repair (LIHR) demonstrated several advantages, including reduced intraoperative blood loss, shorter hospital stays, and quicker return to normal activities compared to open repair (8, 41). The study identified LIHR as an independent factor in reducing postoperative pain, consistent with previous research showing minimal chronic pain following laparoscopic repair (42).

A crucial factor is the anesthetic requirement, LIHR generally requires general anesthesia, but OIHR may be conducted under local or regional anesthesia, rendering it more acceptable for patients who are not candidates for general anesthesia (43). In our cohort, all laparoscopic repairs (100%) were executed under general anesthesia, whereas the majority of open repairs (86.6%) were carried out under local or regional anesthesia. No postoperative cardiovascular problems were seen in either group (0% vs. 0%). This finding contrasts with earlier reports suggesting that regional anesthesia in elderly patients may increase the risk of cardiac and thromboembolic events (16). In addition other extensive research, such as Mayer et al. (2016), which indicated that cardiovascular or thromboembolic problems occurred in 1.2%–1.8% of older patients having inguinal hernia surgery with regional anesthesia (17). Wang et al. (2024) recently showed that ultrasound-guided local nerve block mitigated cardiopulmonary impairment, with cardiovascular events occurring in less than 1% of older individuals (44). In contrast to the stated rates, our result of 0% problems indicates that thorough preoperative cardiac assessment and meticulous anesthetic selection may have enhanced perioperative safety in our patient cohort, and maybe due to small sample size.

This study has limitations inherent to its retrospective, single-center design. The modest sample size (*n* = 384), reliance on medical-record abstraction, and involvement of multiple surgeons may introduce measurement variability and limit external validity. Importantly, the surgical approach (open vs. laparoscopic) was determined by clinical judgment rather than randomization; therefore, selection bias (confounding by indication) is possible. Although we adjusted for age, ASA class, hernia characteristics, anesthesia type, and major comorbidities, residual confounding from unmeasured factors cannot be excluded. In addition, 12-month follow-up may underdetect late recurrences and long-term pain outcomes. These constraints support the need for prospective, multicenter studies ideally randomized or using propensity-based methods with longer follow-up and standardized complication grading.

## 6 Conclusion

This study confirms that laparoscopic inguinal hernia repair provides superior postoperative outcomes compared to the open approach in patients aged 65 years and older, including reduced complication rates, decreased chronic pain, and shorter hospital stays. Key determinants of surgical outcomes were hernia sac size, anatomical location, and anesthesia type, with complex hernias requiring advanced surgical expertise and individualized anesthetic planning. Although laparoscopic repair was associated with longer operative times, open surgery emerged as an independent risk factor for chronic postoperative pain.

From a clinical governance perspective, these findings support the integration of laparoscopic repair as the preferred approach for eligible elderly patients into institutional surgical guidelines. Hospitals could consider adopting structured preoperative assessment protocols to optimize patient selection particularly evaluating comorbidities, anesthetic tolerance, and hernia complexity to ensure both safety and effectiveness. Furthermore, given the long-term benefits for patient recovery and reduced resource utilization, investment in laparoscopic training and equipment may align with broader hospital policy goals of improving surgical quality metrics and cost efficiency.

Future multicenter prospective studies with larger elderly cohorts will be valuable for refining these recommendations and potentially informing national guidelines on inguinal hernia management in this high-risk population.

## Data Availability

The original contributions presented in the study are included in the article/Supplementary Material, further inquiries can be directed to the corresponding author.
